# State Medical Associations

**Published:** 1849-09

**Authors:** 


### Part 3.—Editorial.

### ARTICLE I.

#### STATE MEDICAL ASSOCIATIONS.

We have received from our obliging and attentive friend, Dr. T. Bullard, a copy of the proceedings of the State Medical Convention of Indiana. The assemblage of delegates was not large; but we hail this Convention as the nucleus of a large and respectable State Medical Society, which will do much to elevate the character of the profession in Indiana. Committees were appointed to report a Constitution for a State Medical Society; to report on the propriety of publishing a State Medical Journal; to report resolutions for the consideration of the Convention; and a Committee on Publication.—Dr. Curran in a brief but able report, recommended the establishment of a State Medical Journal. We believe it was in contemplation to establish one immediately, but we have since learned from our friend Dr. Brown, that this project has been postponed.

Among the resolutions reported to the Convention we notice the following:

*Whereas*, The Medical profession in general have been accustomed heretofore to bestow professional services upon clergymen, and their families, gratuitously, and have considered it a pleasure so to do; *And*, *Whereas*, There is no class of our fellow-citizens, with the same influence at command, who so readily countenance quackery and patent medicines, both by signature and influence; therefore,

*Resolved*, That we will hereafter demand remuneration from all clergymen, as from other patients, who, in any manner, are known to lend their influence to irregular practitioners, or to the dissemination of nostrums.

With regard to this subject a friend has suggested to us that there is no propriety in putting clergymen on the "free list," unless it be those who are in straitened circumstances. There is no reason why a person who receives his thousands a year should have the gratuitous services of a physician who perhaps does not receive half as much. We are sorry to see any breach made between the Medical and Clerical professions, between which it is important that the most harmonious relations should exist.

The officers for the ensuing year are: Pres., Dr. Cornet, of Ripley Co.; Vice-Presidents, Drs. Clapp, of New Albany, Johnson, of Cambridge, Dunlap, of Indianapolis, Farquher, of Wabash; Sec's., Drs. Bobbs and Hunt, of Indianapolis; Cor. Sec., Dr. Bullard, of Indianapolis; Treasurer, Dr. Mothershead, of Indianapolis; Librarian, Dr. Jemison of Indianapolis; Delegates to the American Medical Association, Drs. Mullen, Sanders, and Mothershead.

We owe an apology, which we cheerfully make---to the Secretary of the Ottawa Medico-Chirurgical Society, for an unintentional omission to notice in our last number, their circular calling the attention of the profession of this State, to the importance of organizing a State Medical Society, and calling a convention for that purpose at Springfield, on the 1st of January next. We are happy to express our approval of, and co operation in this project; but we doubt the propriety of the time appointed. The close of navigation at that time will have the effect of procuring but a meagre attendance.--- As the matter has been so long deferied, we would suggest that the Convention be postponed until May, at which time we suppose a tolerably full attendance might be procured. We will be glad to hear from the various Medical Societies, and individual members of the profession, their opinions on this subject; and if it is thought best to hold a Convention in January, we will publish a notice to that effect in our next No. We cheerfully accord to our Ottawa friends the praise of being the first to move in a matter of so much interest and importance to the profession in Illinois.

We have received a copy of the proceedings of the Wisconsin State Medical Society, but as it was sent to us in a newspaper, which has been mislaid, we are unable to give our readers any synopsis of the transactions of the Society. We will be much obliged to the Secretary if he will furnish us a fuller copy from the records. The fact of the organization of a State Medical Society in this young and thriving State, affords gratifying evidence that our profession is keeping pace with the rapid improvement of the business and wealth of what will soon be the *great* State of Wisconsin. We wish our *Badger* brethren great success, because we believe they deserve it.

We have also received a copy of the transactions of the Ohio State Medical Society. This is a flourishing institution, and one to which the profession in that State may point with commendable pride. The meeting for the present year quite equalled in interest that of any preceding year.

While on the subject of Medical Associations, we will take the opportunity of presenting to our friends of the Rock River Society our thanks for a copy of their proceedings, an abstract of which will be found on a preceding page. These, we think, will be perused with interest by our readers.

